# Impact of Nursing Leadership Styles on the Staff Turnover Intention in Saudi Arabia: A Cross-Sectional Study

**DOI:** 10.7759/cureus.70676

**Published:** 2024-10-02

**Authors:** Sharifa M Alasiry, Fawzah S Alkhaldi

**Affiliations:** 1 Department of Nursing, Majmaah University, Al Majma'ah, SAU; 2 Department of Nursing Education, Hafar Al-Batin Central Hospital, Ministry of Health, Hafar Al-Batin, SAU

**Keywords:** leadership styles, nurse retention, nurse turnover, nursing leadership and management, registered nurse

## Abstract

Background and objectives

The efficiency of healthcare systems and nursing care depends on appropriate leadership. The purpose of this study was to determine the association between leadership styles of nurse managers and the staff turnover intention in Saudi Arabia. The nurses' intention to quit their jobs was considered as indicative of staff turnover intention.

Methods

A quantitative cross-sectional questionnaire-based study was conducted among 279 nurses, employed in three hospitals in Hafar Al-Batin City, Saudi Arabia. The questionnaire comprised three parts to determine the association between leadership styles of nurse managers and staff turnover intention, and how they related to participants' demographic variables (gender/age/education/income/marital status/experience). The first part collected demographic data, and the second part was an adapted and validated version of the multifactor leadership questionnaire (MLQ-6S) used to assess the perceived leadership styles of nurse managers, based on 21 items. The third part, comprising seven items, was used to assess nurses’ turnover based on intention to quit. The second and third parts of the questionnaire were scored using a five-point Likert scale. Collected data were tabulated and analyzed using a statistical software package for descriptive and inferential statistics (t-test, one-way analysis of variance (ANOVA), and correlation), comparing dependent outcome variables against independent demographic variables. Statistical significance was assumed when p-value was less than 0.05.

Results

Based on leadership style scores, transactional (13.32 out of a maximum of 24; 55.48%) and transformational (26.58 out of a maximum of 48; 55.37%) leadership were the most frequently perceived leadership styles among nurse managers. Laissez-faire leadership (6.31 out of a maximum of 12; 52.57%) was the least frequently perceived style. Upon comparing perceived leadership styles of nurse managers against demographic variables, no significant differences were observed for transformational leadership. However, income and experience of nurses showed significant relationship with the perceived transactional and laissez-faire leadership style scores, respectively. Based on perceived "intention to quit" scores, majority of the nurses expressed uncertain intention to quit (n = 193; 69.2%) and there was a significant yet weak negative correlation between the perceived leadership styles and intention to quit. The correlation coefficient (Pearson’s-r) was lowest for laissez-faire leadership (-0.258) followed by transactional (-0.295) and transformational styles of leadership (-0.348).

Conclusions

The present study indicated a greater degree of transactional and transformational leadership styles among nurse managers than laissez-faire leadership style. While most of the nurses mentioned an uncertain intention to quit, correlating it with the perceived leadership styles it was found that transformational leadership among nurse managers indicated greater retention of nurses within their present jobs. The present research findings underline the importance of prioritizing and fostering healthy workplace environment by the nurse managers, through inculcation of transformational leadership practices. Furthermore, focused, multi-centric studies from around the world can help understand the leadership practices of nursing leaders and its relationship with the intention of nurses to quit their respective roles.

## Introduction

Nurses constitute a major part of the workforce in healthcare facilities providing essential medical management. In recent years, owing to the prevalence of epidemics, the increase in chronic illnesses, and constant unrest around the world, there has been a growing demand for nurses. Despite this unwavering demand, there has always been a shortage of registered nurses around the world [1]. As a result, transformations have been proposed in the structure and functioning of healthcare administrations, in the hope of meeting the anticipated increase in the requirement of registered nurses [2]. Employees including doctors, nurses, and administrators form a crucial part of any healthcare organization and in turn, are responsible for the quality of patient care and healthcare operations [3]. According to the WHO, within the next decade, there will be a growing demand for healthcare workers out of which 50% will be nurses [1]. Under such circumstances, it is imperative that nursing staff turnover and their intention to quit their jobs is given due consideration [4].

In general, the nurses’ intention to quit their profession is a matter of substantial global concern, which could potentially affect the quality of healthcare provided to patients [5]. One of the primary reasons why individuals consider leaving the nursing profession is because they are required to follow instructions from their nurse managers, administrative officers, or doctors [6]. Although there are various other factors like excessive workload and unhealthy work environment for quitting the nursing profession, poor leadership style is most often identified as the major contributor [7]. This is further compounded in the Saudi Arabian healthcare sector, wherein organizational and job-based factors greatly influence burnout among nurses [8].

Over the last decade, the Saudi healthcare system has experienced serious challenges due to the shortage of skilled medical workers [9]. Further, staffing costs are increasing as a direct consequence of the present recruitment system and progressive standards towards nationalizing jobs [10]. Based on recently released data by the Saudi Arabian Ministry of Health, more than 50% of all nurses working in the country are expatriate workers [1]. This dependency on the expatriate workforce has significant ramifications for both the quality and cost of healthcare in the country [9]. Despite governmental efforts to increase impetus towards nursing education and increase the number of nurses entering the job market, it might be a few more years before the majority of nurses in Saudi Arabia are from the local workforce [1]. In this scenario, currently employed nurses deciding to quit their jobs is a problem of serious concern. This further signifies the importance of exploring indicators that determine the nurse’s quality of life in their work environment and determine factors responsible for precipitating their intention to quit their jobs [9].

Evidence-based research reported that relationship-oriented leadership styles, in particular transformational leadership, are effective in increasing staff retention and recruitment by improving nurses’ satisfaction and promoting a healthy working environment [11]. The principle of transformational leadership is based on tenets such as encouraging employees for their performance, inspiring them to achieve meaningful development, and motivating them to perform to their best abilities [11]. On the other hand, the transactional leadership style focuses on the exchange of knowledge, skills, and resources between the leaders and those working under them [10]. While the transformational leader would exert his or her influence over subordinates through encouragement and inspiration, a transactional leader shall do so through motivation based on the system of rewards and/or penalties [11-14]. Since transactional leadership, prioritizes recognition, reward, or punishment, the corrective measures taken by leaders are the defining characteristics of this performance management system [15]. Laissez-faire leadership is a subsection of transactional leadership, which is characterized by unaccountability to anything or anybody with unrestricted discretion over decision-making when leaders are not present [16].

Globally, a close correlational relationship has been reported between the different leadership styles of nurse managers and the levels of work-related stress among nurses and their turnover intentions [17]. Especially, nurses’ turnover rate was found to be higher with transformational leadership than when compared to transactional leadership [14]. However, there is a paucity of research-based evidence reporting on the effect of leadership styles of nurse managers on nurses’ turnover intention in Saudi Arabia. Therefore, the objective of this study was to determine the association between the perceived leadership styles of nurse managers and the staff turnover intention in the Hafar Al-Batin region of Saudi Arabia. The nurses' intention to quit their jobs was considered as an outcome measure indicative of staff turnover intention.

## Materials and methods

Study setting, study design, sampling frame, and sample size

Ethical approval for the present study was obtained from the Institutional Ethics Committee (IRB approval no.: H-05-FT-083, Ministry of Health, Hafar Al-Batin, Saudi Arabia). This research was designed as a quantitative cross-sectional questionnaire-based study to determine the association between nurses’ perceived leadership styles of their nurse managers and their self-perceived intention to quit the present job.

The setting for the study included nurses employed in three main tertiary care hospitals situated in a major city in the north-eastern part of Saudi Arabia and the study timeframe was from September 2023 to December 2023. All nurses working in the aforementioned hospitals and continuously employed in the same hospital for at least one year were included in the sampling frame. Nurses holding administrative positions including nursing supervisors, managers, and head nurses were excluded from the study. A total of 855 nurses comprised the final sampling frame in the three hospitals (King Khalid General Hospital - 345 nurses; Regional Maternity and Children Hospital - 339 nurses; and Hafar Al-Batin Central Hospital - 171 nurses). The sample size for the present study was estimated as 265 participants, with the help of “Steven Thompson Formula” [18], and based on the following assumptions: study population (N) = 855; proportion of error (d) = 0.05; probability percentage (p) = 50%; and confidence level at 95% (z) = 1.96. The final sample size was calculated as 279 participants after a 5% overestimation for incompleteness of responses.

\[ n=\frac{N \times p(1-p)}{\left[\left(N-1 \times\left(\frac{d^2}{z^2}\right)\right)+p(1-p)\right]} \]

\[ n = \frac{855 \times 0.50(1-0.50)}{1500-1 \times \left( \frac{0.0025}{3.84} \right) + 0.50(1-0.50)} \approx 265 \]

\[ n = 265 + 5\% \approx 279 \]

The study participants were recruited using a convenience random sampling technique, and care was exercised to exclude nurses with less than a year’s experience and those holding administrative positions. The questionnaire link was sent through email to all the nurses in the sampling frame and responses were collected on a “first come, first recorded” basis until the required sample size was achieved. In order to ensure equal distribution of samples from the three participating hospitals, study participants were sequentially selected from one hospital after the other. All the respondents to the questionnaire digitally signed a consent declaring their voluntary participation in the study, along with an assurance towards ethical conduct of the study and non-disclosure of participant identifying information.

Study instrument

The study instrument used for data collection was a three-part, self-reported questionnaire, designed in English language. The first part of the questionnaire comprised questions regarding the demographic characteristics of the participants, namely gender, age group, level of education, marital status, professional experience, and income.

The second part of the instrument was aimed at evaluating the leadership styles of the nurse managers, as perceived by the nurses. This was designed based on the "Multifactor Leadership Questionnaire (MLQ)", as designed by Avolio and Bass (1995) and subsequently modified to be called the MLQ-6S [19,20]. The MLQ-6S is a validated questionnaire comprising 21 items and has reportedly been used in studies, both in English and translated languages, to determine leadership characteristics of respondents within the domains of transformational, transactional, and laissez-faire leadership styles [10,14,20,21]. For the present study, the MLQ-6S questionnaire in English was adapted to accommodate the participating nurses’ ability to perceive and report on the leadership styles of their nurse managers. Participants responded to the 21 items based on a "five-point" Likert scale to determine how often their nurse managers engaged in a particular leadership style (0 ‐ not at all; 1 ‐ once in a while; 2 - sometimes; 3 - fairly often; 4 - frequently, if not always). In order to test the clarity, validity, and reliability of the adapted MLQ-6S questionnaire, a pilot study was conducted among 30 volunteer nurses. The results of the pilot study indicated a high degree of reliability and internal consistency for the overall questionnaire, with a Cronbach’s alpha value of 0.912.

The third part of the questionnaire was designed to evaluate the nurses’ intention to quit their present jobs. This was based on seven items reproduced from the study reported by Hand [22], and scored on a "five-point" Likert scale (1 ‐ strongly disagree; 2 ‐ disagree; 3 - neutral; 4 - agree; 5 - strongly agree). Based on the sum of the individual scores for the seven items, an "intention to quit" score was calculated with a possible range from 7 to 35. While scores less than or equal to 15 indicated a low intention to quit, scores between 16 and 25 were regarded as uncertain intention and any score equal to or greater than 26 was indicative of a strong desire to quit the job.

Data collection and statistical analysis

Response data to the questionnaires were collected electronically using a digital survey methodology (Google Forms, Alphabet Inc., Menlo Park, CA, USA). The responses were exported on spreadsheet software and coded for statistical analysis using a statistical software package (Statistical Package for the Social Sciences (IBM SPSS Statistics for Windows, IBM Corp., Version 21.0, Armonk, NY)). The data collection and statistical analysis focused on determining the association between demographic characteristics (independent variables) and the perceived leadership traits of nurse managers and the intention to quit their jobs (dependent variables).

Descriptive statistics (frequencies, measures of central tendency, and dispersion) were calculated for all variables. In order to compare the association between the independent and dependent variables, either an independent t-test or one-way analysis of variance (ANOVA) was used, assuming a 95% statistical significance level (p-value < 0.05 was considered statistically significant). Furthermore, a statistical correlation between the two dependent variables to evaluate the effect of leadership traits of nursing managers on the nurses’ intention to quit their jobs was done.

## Results

Out of the 279 participating nurses, the majority of the nurses were females (n = 188; 67.4%). The mean age of the participants was 31.18 ± 6.19 years (range 19-50 years). The majority of the nurses who participated in the study had completed their bachelor’s education in nursing (n = 136), followed by a diploma (n = 92) and master’s or doctorate (n = 51). While most of them were married (n = 132), only a minority were either single (n = 98) or were divorced or widowed (n = 49). Although only a quarter of the participants reported an experience greater than 10 years (n = 70), more than half of them were reportedly drawing a salary between 5000 and 10000 Saudi Riyals (n = 146). The overall internal consistency for the questionnaire responses had a Cronbach’s alpha value of 0.903. The detailed demographic distribution of the study participants is elucidated in Table 1.

**Table 1:** Demographic characteristics of the study participants and the statistical differences in perceived leadership styles of nurse managers and the nurses' intention to quit scores based on the demographic variables (N = 279). * indicates a significant difference between the groups. Statistical differences based on demographic variables were analyzed using an independent t-test for gender and one-way analysis of variance (ANOVA) for age group, level of education, marital status, income, and experience.

Demographic variable	Variable sub-group(s)	N (%)	Differences in the perceived leadership style of nurse managers and nurses' intention to quit scores based on demographic variables							
			Transformational	p-value	Transactional	p-value	Laissez-faire	p-value	Intention to quit	p-value
Gender	Male	91 (32.6)	26.24 ± 8.24	0.64	13.05 ± 4.57	0.52	6.15 ± 2.78	0.49	21.55 ± 5.19	0.08
	Female	188 (67.4)	26.74 ± 8.68		13.44 ± 4.72		6.38 ± 2.49		20.29 ± 5.93	
Age group	≤30 years	151 (54.1)	26.09 ± 8.38	0.35	12.82 ± 4.63	0.08	6.21 ± 2.57	0.75	20.38 ± 5.78	0.59
	31-40 years	107 (38.4)	26.83 ± 8.79		13.68 ± 4.75		6.43 ± 2.55		21.01 ± 5.59	
	≥41 years	21 (7.5)	28.86 ± 8.07		15.01 ± 4.06		6.44 ± 2.98		21.38 ± 6.07	
Level of education	Diploma	92 (32.9)	25.89 ± 9.75	0.06	12.78 ± 4.95	0.35	5.92 ± 2.83	0.06	20.33 ± 6.76	0.74
	Bachelors	136 (48.7)	27.75 ± 8.59		13.71 ± 4.91		6.71 ± 2.52		20.85 ± 5.46	
	Masters/PhD	51 (18.4)	24.71 ± 4.82		13.25 ± 3.23		5.94 ± 2.17		20.96 ± 4.26	
Marital status	Single	98 (35.1)	26.48 ± 9.12	0.32	13.33 ± 4.86	0.58	6.64 ± 2.53	0.19	20.92 ± 5.92	0.46
	Married	132 (47.3)	27.22 ± 8.61		13.53 ± 4.85		6.23 ± 2.68		20.88 ± 5.61	
	Divorced/Widowed	49 (17.6)	25.06 ± 6.72		12.71 ± 3.71		5.84 ± 2.39		19.78 ± 5.64	
Income	<5,000 SAR	48 (17.2)	24.71 ± 6.92	0.14	11.81 ± 4.29*	0.045	5.75 ± 2.48	0.26	19.42 ± 4.71*	0.014
	5,000-10,000 SAR	146 (52.3)	26.52 ± 7.29		13.73 ± 4.19*		6.42 ± 2.41		20.29 ± 5.17*	
	>10,000 SAR	85 (30.5)	27.74 ± 9.89		13.46 ± 5.47		6.44 ± 2.91		22.13 ± 6.82*	
Experience	1-5 years	83 (29.7)	26.08 ± 9.32	0.67	12.87 ± 4.91	0.19	6.19 ± 2.68	0.03	20.34 ± 6.22	0.32
	5.1-10 years	126 (45.2)	27.08 ± 7.41		13.88 ± 4.33		6.71 ± 2.35*		20.44 ± 5.56	
	>10 years	70 (25.1)	26.27 ± 9.43		12.83 ± 4.90		5.71 ± 2.78*		21.59 ± 5.37	

Analyzing the individual scores for each questionnaire item, as perceived by the participant nurses, the mean values ranged from 1.95 to 2.39 (minimum - 0; maximum - 4) (Table 2). Interestingly, both the highest and lowest mean values were observed for items signifying transactional leadership traits, namely the questionnaire items, “Feels satisfied when we fulfill standards that were agreed upon” and “Does not prefer change as long as things are working as it is”, respectively. Within the transformational leadership traits, ability of nurse managers to help find purpose and meaning for actions (2.32), encourage to improve (2.32), and let others know of the nurses’ opinions and thoughts (2.31) had the highest mean values. The lowest mean value among transformational leadership traits was observed for the item “Encourages us to rethink ideas which we never questioned before” (2.01). For the laissez-faire leadership traits, there were only three questionnaire items and their mean values were 2.01, 2.12, and 2.18 (Table 2).

**Table 2:** Mean values of the individual questionnaire items and leadership style-based scores of the nurse managers, as perceived by the study participants (N = 279).

Perceived leadership styles of nurse managers			Leadership style overall score in mean ± S.D. (range)
Leadership style	Questionnaire items	Mean ± S.D.	
Transformational	Makes others feel good about us	2.17 ± 1.06	26.58 ± 8.52 (0-48)
	Makes others have faith in our actions	2.31 ± 1.16	
	Makes others take pride in associating with us	2.24 ± 1.09	
	Expresses in simple words about what we can and should do	2.14 ± 1.11	
	Provides visualization of whatever we could do	2.24 ± 1.17	
	Helps us find purpose and meaning in our actions	2.32 ± 1.14	
	Enables us to solve old problems by thinking in new directions	2.14 ± 1.13	
	Provides us with new ideas to look at challenging objectives	2.18 ± 1.16	
	Encourages us to rethink ideas which we never questioned before	2.05 ± 1.11	
	Helps and encourages us to improve ourselves	2.32 ± 1.14	
	Let others know of our opinions and thoughts about them	2.31 ± 1.09	
	Gives personalized attention to those of us who feel rejected	2.18 ± 1.18	
Transactional	Tells us what we need to do to be rewarded for our work	2.31 ± 1.14	13.32 ± 4.66 (0-24)
	Provides awards/accolades when we reach our goals	2.18 ± 1.16	
	Calls attention about awards we can get for our accomplishments	2.27 ± 1.14	
	Feels satisfied when we fulfill standards that were agreed upon	2.39 ± 1.09	
	Does not prefer change as long as things are working as it is	1.95 ± 1.19	
	Tells us the standards we need to know to perform our work	2.21 ± 1.14	
Laissez-faire	Let’s continue with our work in the same ways as before	2.18 ± 1.09	6.31 ± 2.59 (0-12)
	Allows us to work in whatever ways that we want to	2.12 ± 1.22	
	Asks us nothing more than things that are absolutely necessary	2.01 ± 1.13	

The sum of scores for questionnaire items pertaining to a given leadership style indicated the perceived scores for that particular leadership style (Table 2). Accordingly, the mean perceived transformational leadership style score of the nurse managers was 26.58 out of a maximum of 48. On the other hand, the mean perceived transactional and laissez-faire leadership style scores were 13.32 (range 0-24) and 6.31 (range 0-12), respectively (Table 2). Based on the leadership style scores it could be deduced that transactional and transformational leadership styles were the most prevalent traits among nurse managers, as they respectively corresponded to 55.48% and 55.37% of the overall scored items, in the MLQ-6S questionnaire. Consequently, laissez-faire leadership trait was the least perceived style among nurse managers, as it comprised only 52.57% of the overall scored items.

Comparing the leadership style-based scores against the independent demographic variables, the mean scores for all leadership styles were higher among female participants than males. Similarly, higher mean scores across the different leadership styles were observed for participants aged 41 years or older, nurses holding a bachelor’s degree, and those with experience between five and 10 years (Table 1). While nurses who were married perceived higher transformational and transactional leadership scores, those who remained single perceived higher laissez-faire leadership scores. In terms of income, nurses earning more than 10000 SAR reported higher mean scores for transformational and laissez-faire leadership styles, and the highest mean transactional leadership score was perceived by nurses having an income between 5000 and 10000 SAR. Statistically significant differences were observed only for transactional leadership scores when compared against nurses’ income, and for laissez-faire leadership scores when compared against nurses’ experience. Nurses earning 5000-10000 SAR perceived significantly higher transactional leadership styles of nurse managers than those earning less than 5000 SAR. Similarly, nurses with experience between 5.1 and 10 years perceived significantly higher laissez-faire leadership style scores than those with more than 10 years of experience (Table 1).

**Figure 1:**
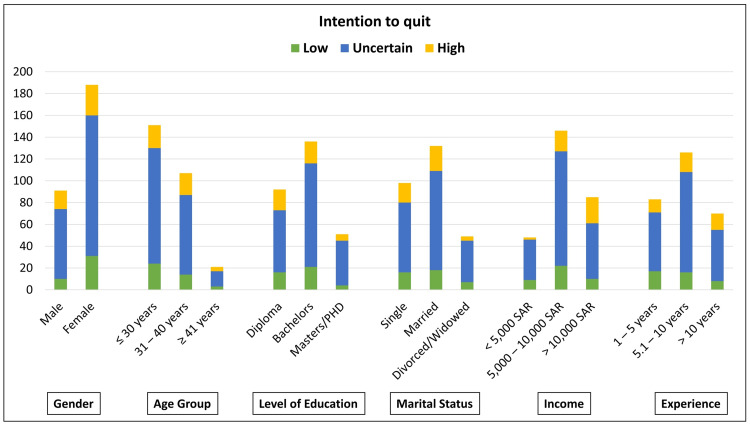
Frequency distribution of participants according to their intention to quit and based on the different demographic variables. Score ranges for "intention to quit": Low - less than or equal to 15; Uncertain - 16 to 25; High - greater than or equal to 26.

The mean values of the individual items in the questionnaire about "intention to quit" ranged from 2.84 to 3.06, indicating an overall intention that varied between disagree and neutral. The mean "intention to quit" score, derived from the sums of the individual questionnaire items, was 20.73 for a range, which varied from 7 to 35 (Table 3). Comparing the "intention to quit" scores against the independent demographic variables, higher mean values were observed among males, participants who were 41 years or older, nurses holding a master’s or doctorate degree, those who were single, nurses with an income more than 10000 SAR and those with experience greater than 10 years. Statistically significant differences in the intention to quit scores were observed only when compared against participant gender and income (Table 1). Where greater mean intention to quit scores were observed among males (21.55 ± 5.19) and those earning income more than 10,000 SAR (22.13 ± 6.82). Overall, a greater "intention to quit" was clearly observed among those with professional confidence in terms of education, experience, and income, and also in participants with favorable personal decision-making abilities such as males, those who were single, and were older than 40 years. Stratifying participants based on their perceived "intention to quit" scores, the majority of the nurses (n = 193; 69.2%) reported an uncertain intention to quit their current job (scores from 16 to 25). This was followed by nurses who reported a high intention to quit (scores ≥ 26; n = 45; 16.1%) and a low intention to quit (scores ≤ 15; n = 41; 14.7%). Interestingly, when distributing the participants with low, uncertain, and high intentions to quit against the demographic variables, the greatest frequency distribution was observed for "uncertain intention to quit" across the respective sub-groups, for all independent variables (Figure 1).

**Table 3:** Mean values of the individual questionnaire items and overall scores for the study participants’ intention to quit from their current jobs (N = 279).

Self-perceived intention to quit		Intention to quit overall score in mean ± S.D. (range)
Questionnaire items	Mean ± S.D.	
I would like to find a comparable job in a different organization	2.94 ± 1.14	20.73 ± 5.72 (7-35)
I will look for a different organization to work for within the next year	2.84 ± 1.21	
I am actively planning to return to school or studies	2.96 ± 1.23	
The results of my search for a new job are encouraging	3.01 ± 1.18	
I will probably look for a new job in the near future	2.97 ± 1.24	
I am currently searching for a job in another organization	2.91 ± 1.21	
I intend to quit	3.06 ± 1.23	

Pearson’s correlation to identify the effect of perceived leadership style scores of the nurse managers on the "intention to quit" scores of the nurses indicated a significant albeit weak negative correlation for all three leadership styles (Table 4). The correlation coefficient (Pearson’s - r) was lowest for the laissez-faire leadership style (-0.258), followed by transactional (-0.295) and transformational leadership styles (-0.348). This indicates a lesser intention to quit among nurses who perceived their nurse managers to have transformational leadership style than among those who perceived their nurse managers to have transactional or laissez-faire leadership styles.

**Table 4:** Correlation between three different perceived leadership style scores of nurse managers and the intention to quit scores reported by nurses (N = 279).

Leadership style of nurse managers	Pearson’s correlation coefficient (r)	p-value
Transformational	-0.348	<0.01
Transactional	-0.295	<0.01
Laissez-faire	-0.258	<0.01

## Discussion

Nurse managers should strengthen interactions and connections with their subordinates by cultivating productive working relationships that are underpinned by loyalty, contributions, professionalism, and mutual respect [13]. These relationships can be sustained by using the least amount of centralization in management and by encouraging the participation of nurses in decision-making [21]. In the long term, this will result in a reduction in the intention of nurses to leave their jobs. The ways in which nurse managers lead their teams in their respective departments play a vital role in determining the rate of nurse turnover. To provide both peer and social support, nurse managers must cultivate a supportive environment in the workplace that encourages staff members to cooperate with one another effectively [12]. It is essential for administrators of healthcare services to help nurse managers in specific departments in learning and practicing the most effective evidence-based leadership techniques [23].

Nurse managers’ leadership practices

The current study reported that transactional (55.48%) and laissez-faire (52.57%) were the most and least common styles of leadership perceived by the nurses. However, transformational leadership was perceived to be practiced by 55.37% of the nurse managers. This result is similar to previous study reports, wherein nurses believed that transactional leadership was the most commonly used style among their nurse managers, followed by transformational leadership [12,14]. Nevertheless, the current findings contradict some of the earlier studies reporting that the majority of nurse managers utilized the transformational leadership style, followed by the transactional leadership style [8,24]. The use of transformational leadership has been the guiding paradigm for educating and mentoring young nurses and student nurses throughout clinical practice [25]. However, nurse managers should adopt both transformational and transactional leadership styles depending on the factors that are present in the working environment [6].

Nurses’ intention to quit

In the present research, approximately 69.2% of the nurses expressed uncertain intention to quit, which was a majority in comparison to the 16.1% of nurses who had a high intention to quit or the 14.7% of nurses who reported low intention to quit. The present values were in line with the findings of Ayalew and Workineh (2020), in which 64.9% of nurses intended to leave their nursing jobs, but without any certainty or predefined timeframe [4]. The present study results were also comparable with previous studies conducted to identify perceived notions of nurses about leaving their current jobs in the Jeddah region of Saudi Arabia (61.5%), Jordan (60.9%), and the Jimma zone of Ethiopia (63.7%) [6,15,26,27]. Having an excessive amount of work to accomplish, being underpaid, having limited possibilities for development, being undervalued, and receiving minimal praise or appreciation from superiors are some of the contributing factors to nurses making a decision to quit [28]. Another interesting aspect noticed in the present study was the significantly higher intention to quit notice among male nurses and those who received an income higher than 10,000 SAR (Table 1). The above findings are in line with those reported by previous studies reporting about nurses' intention to quit and nursing staff turnover in Saudi Arabia [1,9,10]. While it is pertinent to note that nurses receiving higher income might be inclined to search for newer job positions based on experience and/or qualification, a higher male intention to quit could be attributed only to demographic circumstances [1].

Nevertheless, the present study results in terms of intention to quit contradict the data obtained in a study conducted at public hospitals in Hong Kong, where only 4.5% of the general nurses and 5% of the emergency ward nurses intended to leave their jobs [29]. On the contrary, the nurses’ intention to quit ranged as much as 33% in Europe, 21% in Australia, 28.7% in Switzerland, 17% in Turkey, and 21.1% in Brazil [28]. Differences in data across these countries, which were relatively lower than that reported from these developing Middle-Eastern and North African regions, could be attributed to the improved nurse recruitment and retention practices in these developed countries [15,28]. The primary focus of these countries are development of contemporary healthcare facilities, initiatives to boost the number of nursing staff members, compensation incentives, options for work with a flexible schedule, and career progression prospects for staff members [28].

Effects of the leadership styles on nurses’ intention to quit

The current study showed that there was a weakly significant effect of the leadership styles of nurse managers on the nurses’ intention to quit. While a relatively lesser intention to leave the profession was observed with enhanced transformational leadership practice, there was an increasing trend towards quitting the job with respect to transactional and laissez‐faire leadership styles. Similarly, studies have reported that transformational leadership style was associated with a reduced turnover intention in comparison to autocratic and laissez-faire leadership styles [23,24].

Although transactional leadership style (55.48%) was the highest perceived among nurse managers in the present study, perceptions towards transformational leadership (55.37%) practices by nurse managers were only slightly lesser. However, based on correlation, transformational leadership style was associated with a greater level of nurses’ intention to continue in their jobs. Accordingly, it is necessary for nurse managers to foster and practice more transformational leadership traits to boost the percentage of working registered nurses and reduce the turnover of nursing staff [15]. These findings were in alignment with a study reporting that nurse managers who utilized transformational leadership styles increased both the positive aspects of the culture of the hospital and the intention of nurses to continue working [30]. In addition, nurse retention was greatest when higher work satisfaction was experienced, through optimized leadership practices by the healthcare administrators [3].

The findings of the present study are congruent with previous findings that demonstrated a concrete association between transformational or transactional leadership styles and reduced anticipation of nursing staff turnover [17]. Both transformational and transactional styles of leadership can reduce the risk of nurses resigning from their current positions [1,17]. Thus, nurse managers should consider combining these styles of leadership to increase employee’s contentment on the job and the overall quality of nursing care [17]. In contrast, one of the studies found that the increased practice of a laissez-faire leadership style has greatly raised the intention of nurses to leave their positions [16].

Limitations

First, the current study used the perceptions of nurses about leadership practices. It could be more beneficial to evaluate leadership practices based on a combination of nurses’ perceptions and assessments of the styles used by nurse managers. Second, convenience random sampling methodology was used in this study, and this could have limited the potential of the study to generalize findings. Third, while the effect of independent demographic variables was only used as a means for statistical comparison in the present study, their multifactorial confounding effect on the dependent variables (perceived leadership styles and intention to quit) was not evaluated. Nevertheless, this study is one among the first to be reported from Saudi Arabia, which determines the association between the nurses’ perceived ideas relating to the leadership styles of their nurse managers and their intent to remain or leave the job.

## Conclusions

The results of the present study indicated a greater degree of transactional and transformational leadership traits among nurse managers when compared to the laissez-faire leadership style. Although the leadership styles of nurse managers were evaluated based on the perceptions of participating nurses, it was possible to determine their respective notions about the intention to quit their jobs. In general, this was predominantly an uncertain intent among most of the participants, irrespective of the perceived leadership style of nurse managers. Based on correlating the leadership traits and intention to quit, transformational leadership traits among nurse managers fostered greater retention of nurses within their present jobs. On the other hand, a greater intent to quit was seen not only among nurses who perceived a transactional or laissez-faire leadership style of their managers, but also among nurses who were males, earned more than 10000 SAR, and had experience greater than 10 years.

The present research findings underline the importance of prioritizing and fostering a healthy workplace environment by the nurse managers. Maintaining and enhancing nursing roles are crucial, but nurses’ perspectives must also be considered at the highest levels of hospital administration. Providing training and incentives to nurse managers in the adoption of transformational leadership behaviors may increase the quality of their lives and the degree of job satisfaction in Saudi hospitals. In the future, focused and multi-centric studies can help understand better the practice of leadership traits of nursing leaders in Saudi hospitals and their relationship with the intention of nurses to quit their respective roles.
